# Does weight gain from time of indication to date of surgery affect outcomes in total knee arthroplasty?

**DOI:** 10.1186/s42836-026-00379-6

**Published:** 2026-05-08

**Authors:** Anzar Sarfraz, Theodor Di Pauli von Treuheim, Garrett Ruff, Braden V. Saba, Farouk Khury, Ran Schwarzkopf, Joshua C. Rozell, Vinay K. Aggarwal

**Affiliations:** 1 NYU Langone Orthopedic Hospital, NYU Langone Health, New York, NY 10003 USA; 2 The Ruth and Bruce Rappaport Faculty of Medicine, Technion – Israel Institute of Technology, 3109601 Haifa, Israel; 3 Division of Orthopedic Surgery, Rambam Health Care Campus, 3109601 Haifa, Israel

**Keywords:** Total knee arthroplasty, Preoperative optimization, BMI, Weight loss

## Abstract

**Background:**

The impact of body mass index (BMI) on outcomes after total knee arthroplasty (TKA) is a highly debated topic. Our study aims to investigate the implications of BMI changes from the day of surgical booking to the surgery date on perioperative and postoperative outcomes.

**Methods:**

We retrospectively reviewed patients who underwent elective, primary, unilateral TKA at an urban academic institution from 2015–2024 with a minimum 90-day follow-up. The cohort was classified into three groups by percent BMI change from surgical booking date to TKA date: Group 1, decrease in BMI; Group 2, 0–5% increase in BMI; and Group 3, > 5% increase in BMI. Propensity-score matching (1:1:1) based on age, gender, BMI at surgical booking, and smoking status was performed; perioperative and postoperative outcomes were compared. Multivariate regression analysis evaluated risk factors for interval change in BMI.

**Results:**

Before matching, 12,990 patients were included, with 39.6% in Group 1, 41.2% in Group 2, and the remaining 19.2% in Group 3. Notably, Group 3 had the longest length of stay (50.3 h vs. 48.6 [1] & 47.1 [2]; *P* = 0.002) and the lowest discharge-to-home rates (88.7% vs. 89.8% [1] & 91.7% [2]; *P* = 0.014). No significant difference was seen in 90-day ED visits, 90-day readmissions, or revision rates. Logistic regression of the pre-match cohort found that prolonged surgical booking delays were associated with decreased all-cause revisions (OR = 0.98; *P* = 0.038), while percent BMI change in this period did not impact revision incidence. Duration of surgical booking delay had no impact on BMI changes in obese patients.

**Conclusion:**

Our study evaluated preoperative BMI change between surgical booking and TKA, finding that most patients (60.4%) gain weight during this time. While patients with significant BMI increases (> 5%) had longer hospital stays and lower discharge-to-home rates, Percent BMI change during this period did not impact all-cause or septic revision incidence.

## Background

The demand for total knee arthroplasty (TKA) continues to rise in the United States, with incidence expected to increase by 139% by 2040 and 469% by 2060 [1]. This increase is partly attributed to rising obesity rates, with annual increases of 0.97% among patients undergoing TKA [2–5]. Obesity is an established risk factor for postoperative complications, including periprosthetic joint infection (PJI), postoperative coagulopathies, and the need for revision surgery [3, 6, 7]. Specifically, while the incidence of PJI remains below 1% in non-obese patients, it reaches 2% in obese patients and continues to rise [3, 8]. It has been reported that morbidly obese patients undergoing TKA face a 3% to 4% higher risk of developing PJI [9]. Obesity is also associated with prolonged operative times, increased hospital length of stay (LOS), higher rates of discharge to rehabilitation facilities, and elevated 90-day healthcare costs [7, 8, 10–12].

Therefore, the American Association of Hip and Knee Surgeons (AAHKS) recommends delaying TKA for patients with a body mass index (BMI) greater than 40, as the functional benefits are outweighed by the elevated risk of complications [13]. Unfortunately, studies examining the effectiveness of such recommendations have reported limited success. Wilson et al., in their observational review of morbidly obese patients pursuing TKA, found that only 19% lowered their BMI below the institutional cutoff of 40 within two years. Among these 19%, only 5% proceeded with TKA, and the remaining 81% either underwent surgery elsewhere, had TKA despite a BMI above 40, or did not undergo TKA at all [14]. Similarly, Springer et al. reported that only 6.9% of 289 patients prospectively followed after TJA denial met the BMI threshold, despite 23.2% attempting lifestyle changes, including bariatric surgery [15].

Concerningly, even patients who successfully lose weight preoperatively often experience postoperative weight gain. For instance, Riddle et al. found that the magnitude of preoperative weight loss was the second most predictive factor, after age, for postoperative weight gain after TJA [15]. Thus, while preoperative weight loss is an admirable goal while trialing conservative management, success rates remain low, and patients often regress postoperatively [16]. is raises concern that patients may present for total knee arthroplasty at a different BMI than when surgery was indicated. Beyond patient optimization, such weight changes have practical implications for surgical planning, particularly as an increasing proportion of TKA procedures are performed in ambulatory surgery centers, where BMI thresholds often determine venue eligibility. Changes in BMI between surgical booking and the day of surgery may therefore affect the operative setting and contribute to last-minute venue changes or cancellations.

While preoperative and postoperative weight changes in TKA have been widely studied, the interval between surgical booking and the date of surgery remains underexplored. This period is clinically meaningful because it represents the point at which patients have already met institutional eligibility criteria, including BMI thresholds, and have been formally booked for surgery. Once a surgical date is secured, patient behavior and motivation may change, yet surgeons often assume weight stability during this final preoperative phase. Prior studies evaluating perioperative weight change typically assess broader preoperative timeframes or postoperative trends and do not isolate this distinct interval. Our study aimed to assess weight changes from the surgical booking date to the surgery date. We sought to determine demographic factors that influence these weight fluctuations and peri-operative outcomes (operative time, length of stay, and discharge disposition) and post-operative outcomes (90-day emergency department visits, 90-day readmissions, infection-related surgical outcomes [irrigation and debridement and septic revision], aseptic revision, and deep vein thrombosis).

## Methods

### Cohort selection and eligibility criteria

This was a retrospective review of all primary, elective TKA procedures between September 1st, 2015, and March 31st, 2024, indicated by a diagnosis of osteoarthritis. Patients aged 18 years and older undergoing a unilateral TKA procedure with at least 90 days of follow-up were included. Patients who underwent a revision TKA, bilateral procedures, or non-elective procedures were excluded. Our institution recommends patients with a BMI > 40 kg/m^2^ delay surgery to lose weight before booking, although our absolute ineligibility threshold is 50 kg/m^2^. Only under extenuating circumstances are patients with BMI > 50 kg/m^2^ indicated for surgery, after all weight loss attempts are fulfilled. Institutional review board approval was obtained before initiating the study: i17-01223.

### Patient characteristics and outcome measures

Baseline characteristics, including gender, age, race, smoking status, and American Society of Anesthesiologists (ASA) class, were collected from electronic medical records. Weight and BMI were recorded at the clinic visit on the date of booking and on the day of surgery. Obesity-related factors, such as diabetes diagnosis, glucagon-like peptide-1 (GLP-1) agonist prescriptions, and history of bariatric surgery, were also documented. Perioperative data included operative time, length of stay (LOS), and discharge disposition. Postoperative outcomes included 90-day emergency department visits, 90-day readmissions, DVT, and rates of septic and aseptic revision.

### Study design

Patient height and weight were obtained at the preoperative clinic visit at which surgical indication and booking occurred, and again on the day of surgery as part of routine clinical care, using calibrated institutional scales operated by trained clinical staff. BMI was calculated using the standard formula of weight in kilograms divided by height in meters squared (kg/m.^2^) [17]. Weight changes were defined as the percentage change in BMI [(BMI at surgery date—BMI at clinic visit)/BMI at clinic visit] and as absolute BMI change [BMI at surgery date—BMI at clinic visit], with positive numbers representing weight gain and negative numbers representing weight loss. Group classification was based on percent BMI change from booking to surgery: Group 1, those who lost weight after surgical booking; Group 2, those who gained some weight (< 5% of their original BMI) after booking; and Group 3, those who gained significant weight (> 5% of their BMI) after booking. A BMI change of 5% was used to stratify patients, as this value is considered clinically significant by the US Food and Drug Administration [18]. Patients’ socioeconomic status (SES) was categorized by quartile of median household income for a patient’s home ZIP code, as suggested by Konopka et al.[19, 20]

**Fig. 1:**
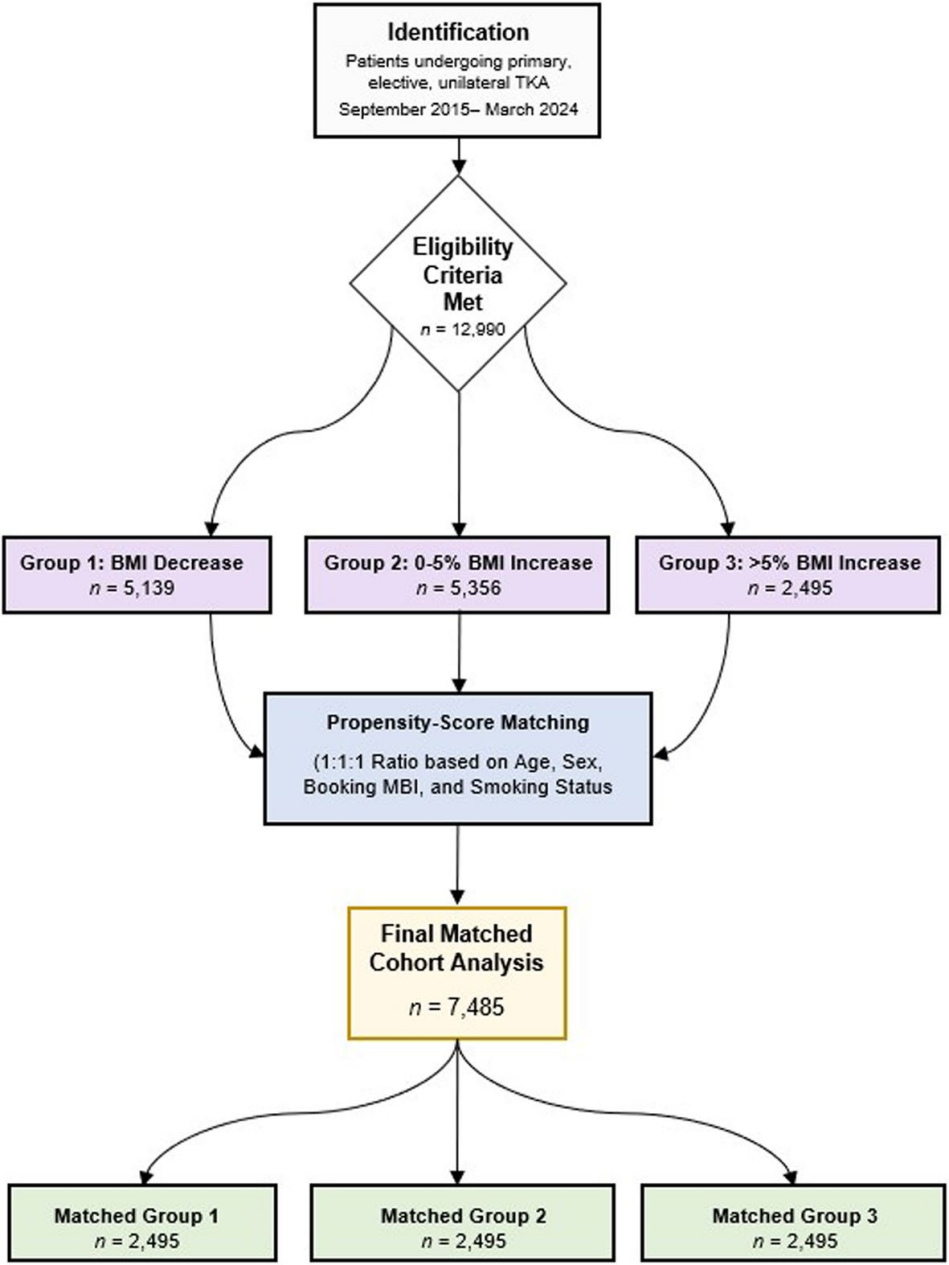
Study flow diagram

Patients were propensity-score matched to Group 3 in a 1:1:1 ratio based on age, BMI on the day of surgical booking, gender, smoking status, ASA class, diabetes history, and socio-economic class. We selected a 1:1:1 matching ratio to create equally sized comparison groups centered on the > 5% BMI gain cohort, which was the primary exposure group. Sequential matching of Group 1 to Group 3, and then Group 2 to Group 3, was performed **(**Fig. 1**)**. Propensity scores were estimated using logistic regression, and pairwise matching of the three cohorts was performed using the Nearest Neighbor method without matching replacement or setting caliper widths. Balance testing resulted in standardized mean differences (SMD) of 0.002 for age, 0.011 for pre-operative BMI, 0.017 for gender, 0.014 for smoking status, 0.024 for ASA class, 0.015 for diabetes history, and 0.035 for SES. SES was included as a proxy for social determinants of health that may influence perioperative outcomes. This match was considered successful as all SMD values were below 0.1. Despite matching and multivariable adjustment, residual confounding from unmeasured variables remains possible.

Multivariable logistic regression analysis was performed on the pre-match cohort to isolate the effect of preoperative BMI change on the incidence of all-cause and septic revisions. Additionally, multivariable linear regression was conducted on the pre-match cohort to identify factors influencing BMI changes from surgical booking to the date of surgery. Multicollinearity was assessed using generalized variance inflation factors, with minimal collinearity identified (the greatest GVIF was 1.33). To further examine the relationship between booking delay and preoperative weight fluctuations, patients were stratified into delay groups of 0–1 month, 1–4 months, and 4–12 months, and median BMI changes were compared.

### Data analyses

Power analysis for multivariate regression analyses was performed using the method proposed by Hsieh et al. [21], to detect an association between percent BMI change and revision incidence. Assuming a two-sided α = 0.05 and 80% power, with the standard deviation of percent BMI change of 5.5 and an odds ratio of 1.05 per 1% BMI change, we require approximately 4467 patients for overall revision (baseline rate 2.5%) and 21,885 patients for aseptic revision (baseline rate 0.5%).

Data were analyzed using the Statistical Package for the Social Sciences version 26.0 (IBM Corp., Armonk, NY, USA). All continuous variables were non-normally distributed as determined with the Anderson–Darling test (*P* < 0.05). Continuous variables were presented as means and standard deviations and compared using Kruskal–Wallis tests. Categorical variables were expressed as counts and percentages, and compared using chi-square tests. A significance threshold of *P* < 0.05 was used.

## Results

### Baseline characteristics

In total, 12,990 TKA patients met our eligibility criteria. Before matching, 39.6% of patients lost weight after booking (Group 1), 41.2% gained some weight (Group 2), and 19.2% gained significant weight (Group 3).

After matching, there were no significant differences between groups in gender, age, smoking status, ASA class, income quartile, prevalence of diabetes, or BMI at the time of surgical booking. The average time from booking date to surgery date was 61 days for all patients (Group 1: 61.1 ± 49.1, Group 2: 57.3 ± 47.8, Group 3: 65.3 ± 52.9; *P* < 0.001). While statistically significant, a difference of one week is clinically insignificant in the context of average delays of nine weeks. Group 2 had the highest proportion of Caucasian patients (*P* < 0.001). Group 3 had the highest BMI on the day of surgery (34.0 vs. 30.2 [1] & 31.8 [2]; *P* < 0.001) (Table 1).

**Table 1:** Baseline characteristics

	**Group 1** ***n* = 2495**	**Group 2** ***n* = 2495**	**Group 3** ***n* = 2495**	***P*-value**
**Sex**				0.608
Men	654 (26.2%)	682 (27.3%)	680 (27.3%)	
Women	1841 (73.8%)	1813 (72.7%)	1815 (72.7%)	
**Age** (years)	67.9 ± 9.2	67.8 ± 9.1	67.9 ± 9.1	0.994
**Race**				** < 0.001**
White	1328 (53.2%)	1429 (57.3%)	1304 (52.3%)	
African American	503 (20.2%)	484 (19.4%)	472 (18.9%)	
Asian	159 (6.4%)	126 (5.1%)	145 (5.8%)	
Other	505 (20.2%)	456 (18.3%)	574 (23.0%)	
**Smoking Status**				0.974
Current	126 (5.1%)	120 (4.8%)	126 (5.1%)	
Former	778 (31.2%)	780 (31.3%)	763 (30.6%)	
Never	1591 (63.8%)	1595 (63.9%)	1606 (64.4%)	
**Weight** (kg)				
Office visit	83.3 ± 17.8	85.1 ± 18.6	85.7 ± 18.4	** < 0.001**
Surgery day	81.3 ± 17.3	85.3 ± 18.7	88.1 ± 19.3	** < 0.001**
**BMI** (kg/m^2^)				
Office visit	31.2 ± 5.7	31.1 ± 5.8	31.1 ± 5.8	0.841
Surgery day	30.2 ± 5.5	31.8 ± 5.9	34.0 ± 6.4	** < 0.001**
**ASA Score**				0.944
1	42 (1.7%)	45 (1.8%)	42 (1.7%)	
2	1368 (54.8%)	1360 (54.5%)	1362 (54.6%)	
3	1053 (42.2%)	1065 (42.7%)	1057 (42.4%)	
4	32 (1.3%)	25 (1.0%)	34 (1.4%)	
**Income Quartile**				0.782
1st (0–25%)	889 (35.6%)	900 (36.1%)	910 (36.5%)	
2nd (25–50%)	768 (30.8%)	788 (31.6%)	761 (30.5%)	
3rd (50–75%)	515 (20.6%)	470 (18.8%)	488 (19.6%)	
4th (75–100%)	323 (12.9%)	337 (13.5%)	336 (13.5%)	
**Diabetes**	558 (22.4%)	559 (22.4%)	581 (23.3%)	0.680
**GLP-1 Agonist use**	119 (4.8%)	110 (4.4%)	88 (3.5%)	0.081
**Bariatric Surgery History**	39 (1.6%)	48 (1.9%)	45 (1.8%)	0.615

Continuous variables are represented as mean [range]; categorical variables are represented as count (percent). Statistically significant *P*-values (*P* < 0.05) are bolded for emphasis. ASA, American Association of Anesthesiology; BMI, Body mass index; GLP, Glucagon-like peptide. Group 1 = > 5% weight loss preop, Group 2 = < 5% weight change preop, Group 3 = > 5% weight gain preop

When assessing differences in obesity-related patient characteristics, GLP-1 agonist prescriptions were most common in Group 1 (4.8%) compared to Group 2 (4.4%) and Group 3 (3.5%), although this was not statistically significant. There was no significant difference in the prevalence of bariatric surgery history between groups (Table 1).

### Perioperative and clinical outcomes

The average LOS was longest in Group 3 (50.3 h) compared to Groups 1 and 2 (48.3 & 48.0 h, respectively; *P* = 0.022). While most patients were discharged home, Group 3 also had a significantly lower discharge-to-home rate (88.7% vs. 89.4% [1] & 91.1% [2]; *P* = 0.027). Mean follow-up duration was similar across cohorts (Cohort 1: 2.2 ± 2.1 years; Cohort 2: 2.2 ± 2.0 years; Cohort 3: 2.1 ± 2.0 years; *P* = 0.593), with an overall mean follow-up of 2.2 years. There were no significant differences among groups in operative time, 90-day emergency department visits, 90-day readmissions, infection-related surgical outcomes (I&D and septic revisions), aseptic revision, or DVT risk (Table 2).

**Table 2:** Perioperative variables and postoperative clinical outcomes

	**Group 1** ***n* = 2495**	**Group 2** ***n* = 2495**	**Group 3** ***n* = 2495**	***P*-value**
**Operative Time** (minutes)	107.4 ± 24.8	107.7 ± 26.5	106.3 ± 24.7	0.143
**LOS** (hours)	48.0 ± 30.7	48.3 ± 31.1	50.3 ± 34.4	**0.022**
**Discharge Disposition**				**0.027**
Home	2231 (89.4%)	2273 (91.1%)	2214 (88.7%)	
SNF	239 (9.6%)	200 (8.0%)	255 (10.2%)	
ARF	22 (0.9%)	22 (0.9%)	26 (1.0%)	
Other	3 (0.1%)	0 (0.0%)	0 (0.0%)	
**90-Day ED visits**	140 (5.6%)	107 (4.3%)	118 (4.7%)	0.087
**90-Day Readmissions**	94 (3.8%)	86 (3.4%)	82 (3.3%)	0.642
**DVT**	18 (0.7%)	13 (0.5%)	15 (0.6%)	0.660
**Mean Follow-up (years)**	2.2 ± 2.1	2.1 ± 2.0	2.1 ± 2.0	0.593
**Infection-Related Surgical Outcomes**				
I&D	26 (1.0%)	22 (0.9%)	15 (0.6%)	0.226
Septic Revision	10 (0.4%)	11 (0.4%)	11 (0.4%)	0.969
Aseptic Revision	52 (2.1%)	42 (1.7%)	58 (2.3%)	0.268

Continuous variables are represented as mean [range]; categorical variables are represented as count (percent). Statistically significant p-values (*P* < 0.05) are bolded for emphasis. LOS, Lengths of hospital stay; SNF, Skilled nursing facility; ARF, Acute rehabilitation facility; DVT, deep vein thrombosis; ED, Emergency department; I&D, irrigation and debridement; PJI, Prosthetic joint infection. Group 1 = > 5% weight loss preop, Group 2 = < 5% weight change preop, Group 3 = > 5% weight gain preop

Multivariate regression analysis showed that the need for all-cause revision decreased with increasing age (odds ratio [OR] = 0.95, *P* < 0.001), Asian race (OR = 0.43, *P* = 0.045), and increased weeks of surgical booking delay (OR = 0.98; *P* = 0.039). Need for all cause-revision increased with Black race (OR = 1.65, *P* = 0.001) and ASA IV status (OR = 6.36, *P* = 0.007). The need for septic revision decreased with increasing age (OR = 0.95, *P* = 0.001) and increased with GLP-1 usage (OR = 2.81, *P* = 0.017). Notably, only 601 patients were using GLP-1 agonists, with eight of these patients (1.3%) undergoing septic revision, compared to 53 patients (0.4%) not using GLP-1 agonists undergoing septic revision. The low incidence of both GLP-1 agonist use and septic revisions limits this analysis. BMI change from time of booking to surgery did not impact all-cause or septic revision rates (Table 3).

**Table 3:** Multivariable logistic regression results for TKA patient characteristics predictive of need for revision, stratified by all-cause revisions and septic revisions

	**All-Cause Revisions**		**Septic Revisions**	
	**Adjusted OR [95% CI]**	***P*-Value**	**Adjusted OR [95% CI]**	***P*-Value**
**Age At Surgery**	0.95 [0.94, 0.97]	** < 0.001**	0.95 [0.92, 0.98]	**0.001**
**BMI At Surgery**	0.99 [0.97, 1.01]	0.178	0.98 [0.94, 1.03]	0.380
**% BMI Change**	1.01 [0.99, 1.03]	0.368	1.01 [0.96, 1.05]	0.665
**Male Sex**	1.21 [0.95, 1.54]	0.118	1.24 [0.72, 2.12]	0.429
**Race**				
White	1.0	-	1.0	-
Black	1.65 [1.23, 2.21]	**0.001**	0.90 [0.42, 1.81]	0.776
Asian	0.43 [0.17, 0.90]	**0.045**	0.32 [0.02, 1.53]	0.269
Other	1.20 [0.88, 1.62]	0.243	0.79 [0.36, 1.59]	0.531
**Surgical Booking Delay** (in weeks)	0.98 [0.96, 0.999]	**0.039**	0.98 [0.94, 1.01]	0.239
**Smoking Status**				
Never	1.0	-	1.0	-
Former	1.27 [1.00, 1.61]	0.052	1.53 [0.90, 2.60]	0.117
Current	1.20 [0.74, 1.86]	0.431	0.93 [0.22, 2.70]	0.907
**ASA Class**				
I	1.0	**-**	1.0	**-**
II	2.44 [0.91, 9.98]	0.132	0.57 [0.16, 3.62]	0.456
III	3.12 [1.14, 12.87]	0.057	1.05 [0.29, 6.77]	0.953
IV	6.36 [1.81, 29.59]	**0.007**	1.93 [0.22, 17.29]	0.528
**Income Quartile**				
1st (0–25%)	1.0	-	1.0	-
2nd (25–50%)	1.05 [0.79, 1.40]	0.721	1.13 [0.55, 2.30]	0.736
3rd (50–75%)	1.02 [0.74, 1.41]	0.897	1.37 [0.66, 2.85]	0.398
4th (75–100%)	1.32 [0.92, 1.89]	0.126	1.64 [0.73, 3.60]	0.221
**Diabetes**	0.93 [0.70, 1.24]	0.633	0.81 [0.40, 1.54]	0.532
**History of Bariatric Surgery**	1.14 [0.55, 2.09]	0.703	2.18 [0.63, 5.71]	0.157
**GLP-1 Usage**	1.04 [0.60, 1.71]	0.883	2.81 [1.13, 6.26]	**0.017**

Statistically significant *P*-values (*P* < 0.05) are bolded for emphasis. ASA, American Society of Anesthesiologists; BMI, body mass index; CI, confidence interval; GLP-1, glucagon-like peptide-1 agonist; OR, odds ratio

### Factors affecting preoperative weight change from booking to surgery

Multivariable regression analysis was performed to evaluate risk factors affecting weight change from booking date to surgery date. Increased BMI at the time of booking (Effect size: − 0.03, *P* < 0.001), male sex (− 0.21, *P* < 0.001), income in the highest quartile (− 0.11, *P* = 0.028), and former smoking status (− 0.09, *P* = 0.006) were associated with decreases in BMI. Age, race, ASA class, and time from booking to surgery did not significantly impact BMI changes. Obesity-related patient characteristics, including a diagnosis of diabetes, history of bariatric surgery, and GLP-1a usage, were also not significantly associated with BMI change from booking to surgery (Table 4).

**Table 4:** Multivariate linear regression analysis of factors affecting absolute BMI change

	**Effect Size [95% CI]**	***P*-Value**
**Age At Surgery**	0.00 [− 0.01, 0.00]	0.132
**BMI At Booking**	− 0.03 [− 0.03, − 0.02]	** < 0.001**
**Male Sex**	− 0.21 [− 0.28, − 0.14]	** < 0.001**
**Race**		
White	-	-
Asian	− 0.12 [− 0.26, 0.02]	0.095
Black	− 0.05 [− 0.14, 0.04]	0.246
Other	0.05 [− 0.04, 0.13]	0.283
**Surgical Booking Delay** (in weeks)	0.00 [− 0.01, 0.00]	0.417
**Smoking Status**		
Never	-	-
Former	− 0.09 [− 0.16, − 0.03]	**0.006**
Current	− 0.03 [− 0.18, 0.12]	0.670
**ASA Class**		
I	-	**-**
II	0.09 [− 0.15, 0.32]	0.456
III	0.22 [− 0.01, 0.46]	0.061
IV	0.21 [− 0.13, 0.56]	0.220
**Diabetes**	− 0.02 [− 0.09, 0.06]	0.671
**History of Bariatric Surgery**	0.09 [− 0.12, 0.31]	0.395
**GLP-1a Usage**	− 0.08 [− 0.23, 0.07]	0.296
**Income Quartile**		
1st (0–25%)	-	-
2nd (25–50%)	0.00 [− 0.08, 0.08]	0.970
3rd (50–75%)	− 0.05 [− 0.14, 0.04]	0.257
4th (75–100%)	− 0.11 [− 0.21, − 0.01]	**0.028**

Effect sizes represent the estimated added (subtracted) BMI change due to the given covariate (e.g., an additional year of age is associated with no effect (0.00 added) on the BMI change from booking to surgery date). Statistically significant *P*-values (*P* < 0.05) are bolded for emphasis. ASA, American Society of Anesthesiologists; BMI, body mass index; GLP-1, glucagon-like peptide-1 agonist

**Fig. 2:**
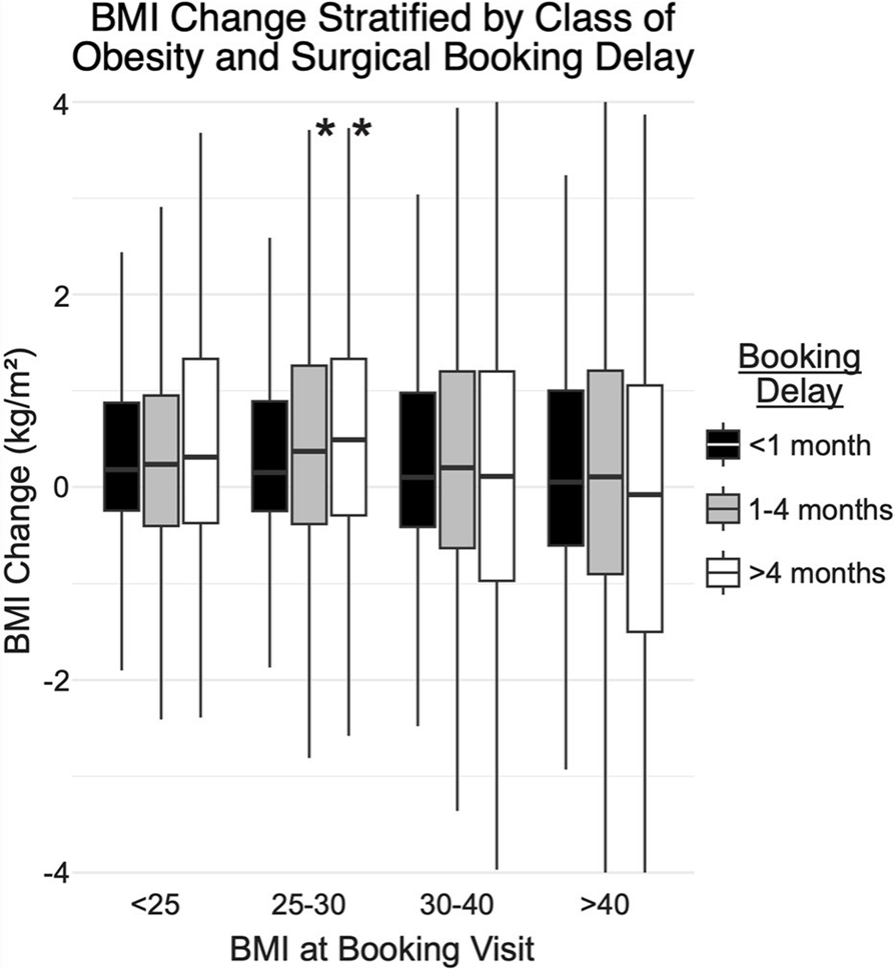
Body Mass Index (BMI) change for patients from the booking date to surgery date, stratified by patient BMI at the time of surgical booking. Kruskal–Wallis and post hoc Dunn tests compare each time point to the first month within each group, with * indicating *P* < 0.05, ** *P* < 0.01, and *** *P* < 0.001

To further assess the impact of booking delay, patients were stratified by BMI at the time of surgical booking and the duration of delay from booking to surgery. In patients with a booking-day BMI between 25 and 30 (classified as overweight), median increases in BMI were significantly greater in those with delays of one to four months (+ 0.40 vs. + 0.17; *P* = 0.011) and greater than four months (+ 0.50 vs. + 0.17; *P* = 0.016) compared to those with delays less than one month. There was no significant effect of booking delay on BMI changes in patients with booking-day BMIs less than 25 or greater than 30 (Fig. 2).

## Discussion

Given current AAHKS guidelines recommending surgical delay in patients with a BMI over 40 kg/m^2^, our study sought to investigate the trends and impact of preoperative BMI changes after patients’ surgical booking dates, the time at which they must meet BMI inclusion thresholds. Our study found that 60.4% of patients gained some weight from their booking date to their surgery date, with 19.2% of patients gaining significant weight (> 5% BMI increase). Patients who gained significant weight had longer hospital stays and lower discharge-to-home rates. However, BMI change between booking and surgery was not associated with 90-day complications or revision rates. Furthermore, while longer delays from booking to surgery were associated with lower odds of all-cause revision in multivariable analysis, this relationship should be interpreted with caution and not assumed to be causal.

We found that 19% of patients undergoing TKA experienced a significant (> 5%) increase in BMI between surgical booking and date of TKA, while an additional 41% gained some weight, and the remaining patients lost weight. The mean time between booking and date of surgery in our cohort was 61 days. These findings are comparable to other TKA studies investigating general trends in preoperative weight fluctuations. Kim et al. assessed TKA patients in the year before surgery, finding that 17% experienced significant (> 5%) BMI increases in the 12 months preoperatively[22]. However, Inacio et al. found that only 7% of TKA patients gained significant (> 5%) weight in the year preoperatively[23]. As our study found that 19% of patients gained significant weight from the time of booking to surgery, which is a similar, if not elevated, rate compared to current literature, this period may represent a time of increased susceptibility to BMI increases for TKA patients. Therefore, while total joint arthroplasty is a motivating factor for lifestyle changes and weight management [24], we find that a majority of patients gain weight after surgical booking, suggesting that continued education and incentivization of weight management during this period may be of particular benefit.

After matching patients to control for demographic factors, we evaluated the perioperative and clinical outcomes of patients based on their preoperative weight fluctuations. Patients who gained significant weight preoperatively (Group 3) had longer LOS and decreased rates of home discharge following TKA. However, clinical outcomes were not different between groups, as there were no differences in 90-day ED visits, readmissions, or revisions. Furthermore, logistic regression analysis assessing the risk of all-cause and septic revisions found no association with preoperative weight fluctuations. Therefore, while many patients gain weight after surgical booking, this weight change may not significantly impact adverse outcomes following surgery, although those patients gaining substantial weight may be at elevated risk of discharge to a rehabilitation facility.

In multivariable logistic regression, longer booking delay was associated with a small reduction in the odds of all-cause revision, while no association was observed with septic revision. This finding should be interpreted cautiously and should not be viewed as evidence that delaying surgery is protective. Several non-causal explanations are possible. Patients who experience longer time interval may undergo additional preoperative medical optimization or risk modification that was not fully captured in the available covariates. Variability in surgeon level scheduling practices and selection factors may have influenced both surgical timing and revision rate. In addition, the longer time interval may reflect patient preference (for example, choosing a later date for personal, work, or other social reasons), and such patients may differ systematically from those who opt for earlier surgery. Finally, revision is a time-dependent outcome, and retrospective analyses are susceptible to time-related biases, including survivorship bias, as only patients who ultimately proceed to surgery and remain in follow-up are included. Given the modest effect size and the secondary nature of this analysis, we interpret this association as hypothesis-generating rather than causal.

While the detrimental effect of obesity on TJA outcomes is well-established [25–28], the effects of preoperative weight change are less clear, particularly from the time of surgical booking to the surgery date. Weight loss is not always clinically beneficial, though. Preoperative weight loss > 5% within nine months of surgery has been shown to be associated with inferior outcomes, including higher PJI and all-cause revision rates in some TKA studies [22, 29]. This may result from potential malnutrition and inflammatory changes due to short-term dieting and weight loss.[30, 31] However, other studies have shown that preoperative weight loss a year before TKA reduces these risks, suggesting that a stable, reduced BMI provides similar benefits to a lower BMI at baseline [22, 29]. Postoperative weight gain, however, has also been linked to inferior outcomes, including higher PJI risk [22]. While prior literature suggests that major weight fluctuations may affect outcomes, our findings indicate that BMI change between booking and surgery affected discharge metrics only and was not associated with postoperative complications or revision risk.

The final objective of our study was to identify predictive factors for weight gain after surgical booking. Importantly, some arthroplasty surgeons are anecdotally concerned that prolonged delays from booking to surgery will predispose patients to weight gain, particularly given the decreased physical function of patients with end-stage arthritis awaiting joint replacement. However, our study found no effect of surgical booking delay on preoperative weight fluctuations. This suggests that delaying surgery for medical or logistical reasons does not inherently result in progressive weight gain, although modest increases after booking remain common. From a clinical standpoint, these findings support continued reinforcement of weight management counseling after surgical booking, rather than assuming weight stability once a surgical date is secured. Overweight patients, in particular, may benefit from closer monitoring during this interval, as they appeared more susceptible to preoperative weight gain in our cohort.

Non-lifestyle strategies for weight optimization are an expanding area of interest in elective surgery, including bariatric surgery and GLP-1 agonist therapy. Bariatric surgery has shown mixed effects on outcomes depending on timing and procedure type prior to TKA, [22, 32] and is associated with its own risks. Recent studies of GLP-1 agonists suggest potential benefits in preoperative weight loss, sustained weight management, and reduced postoperative complications. [33] While our study did not specifically evaluate the effectiveness of these interventions, GLP-1 use was least prevalent among patients with significant weight gain and most common among those who lost weight. Taken together, our findings suggest that surgeons can individualize surgical timing without undue concern for worsening postoperative outcomes from modest weight gain, while continuing to emphasize targeted optimization strategies for patients at higher risk of preoperative weight increase.

## Limitations

This study has several limitations. First, as a retrospective observational analysis, it is subject to selection bias and potential misclassification bias. Height and weight were measured using standardized institutional equipment but were obtained as part of usual clinical care rather than a research protocol. As a result, small variations related to time of day, clothing, or hydration may have occurred. However, measurement practices were consistent across patients and timepoints, making systematic bias unlikely. Because only patients who ultimately proceeded with surgery were included, selection bias related to surgical candidacy may be present. In addition, reliance on electronic medical record documentation may introduce outcome misclassification. Patients with missing key BMI data were excluded from analysis, and missingness among included covariates was minimal. In addition, we defined clinically meaningful weight change using a threshold of greater than 5% BMI change, which exceeds expected measurement variability and has been widely used in arthroplasty research [18, 22]. Our cohort was limited to elective, primary, unilateral TKA, as this was the focus of the study. Therefore, the findings may not be generalizable to bilateral, revision, or non-elective procedures.

Second, the study included patients across a broad range of baseline BMIs. Patients with lower baseline BMI may be more sensitive to relative weight changes than those with higher BMI. To address this, we matched patients based on BMI at the time of surgical booking and performed analyses stratified by baseline BMI, allowing us to account for differences in weight change behavior across BMI categories. At our institution, patients with BMI > 40 kg/m^2^ are generally recommended to lose weight before booking, and those with BMI > 50 kg/m^2^ are considered for surgery only under rare extenuating circumstances. These thresholds are not absolute and may vary between surgeons in our large tertiary care center. Because our cohort includes only patients who ultimately had TKA, we could not capture patients who were deferred based on BMI alone, which may limit generalizability.

Third, several patient-level factors that may influence postoperative recovery were not consistently available, including preoperative functional status, nutritional markers, detailed comorbidity indices, and measures of frailty or activity level. Although ASA class, diabetes, bariatric surgery history, and GLP-1 use were included in regression models to account for comorbidity burden and medication exposure, additional comorbidities and medications were not uniformly available and may represent residual confounders. We were able to account for socioeconomic status using median household income by ZIP code; however, some residual confounding from unmeasured clinical and functional factors may remain. Importantly, the primary aim of this study was to evaluate short-term BMI changes between surgical booking and surgery, rather than to develop a comprehensive model of postoperative risk.

Fourth, septic revision after primary total knee arthroplasty is a relatively rare event, with contemporary registry and administrative data reporting incidence rates ranging from approximately 0.5% to 2% in modern practice [34–36]. Given the low absolute event count in our cohort, analyses evaluating septic revision may be underpowered to detect small effect sizes, and the absence of a statistically significant association should be interpreted cautiously.

Finally, data on hyperlipidemia and lipid-lowering medication use were not available for inclusion in the analyses. Prior arthroplasty literature has evaluated dyslipidemia as a component of metabolic comorbidity burden associated with perioperative risk, and studies assessing lipid-lowering therapy in TKA typically define exposure as chronic over extended preoperative periods rather than short, time-varying intervals [37]. The primary objective of this study was to evaluate the impact of percent BMI change between surgical booking and surgery, rather than baseline metabolic comorbidity burden. Because hyperlipidemia is a relatively chronic condition and unlikely to change meaningfully during the booking-to-surgery interval, its omission is unlikely to substantially confound the observed associations between booking-to-surgery interval weight change and perioperative or postoperative outcomes. Nonetheless, future studies incorporating lipid profiles and medication use may further clarify how metabolic health interacts with preoperative weight trajectories.

## Conclusions

Preoperative weight loss remains an essential component of patient optimization in TKA, although the incentive to lose weight may diminish once patients obtain a surgical booking date. Our study provides a unique perspective on patients’ weight fluctuations from their surgical booking date to the date of surgery, finding that 19% of patients had significant BMI increases (> 5%) during this period. Such weight gain was associated with extended hospital stays and lower discharge-to-home rates but was not associated with significant differences in postoperative complications or revision rates in this cohort.

Baseline BMI influenced preoperative weight trajectories, as patients with BMI 25–30 kg/m^2^ demonstrated greater susceptibility to weight gain with longer booking to surgery delays, a pattern not observed in patients with BMI < 25 or greater than 30. These findings suggest that baseline BMI influences preoperative weight trajectories and may help identify patients who could benefit from targeted counseling during prolonged preoperative waiting periods.

Arthroplasty surgeons should anticipate weight gain among TKA patients after booking, although the clinical impact of this weight gain appears limited to discharge planning metrics within the scope of this study.

## Data Availability

No datasets were generated or analysed during the current study.

## Abbreviations

AAHKS: American Association of Hip and Knee Surgeons
ASA: American Society of Anesthesiologists
BMI: Body mass index
ED: Emergency department
GLP-1: Glucagon-like peptide-1
LOS: Length of stay
PJI: Periprosthetic joint infection
SMD: Standardized mean difference
TJA: Total joint arthroplasty
TKA: Total knee arthroplasty

## References

[1] Shichman I, Roof M, Askew N, Nherera L, Rozell JC, Seyler TM, et al. Projections and epidemiology of primary hip and knee arthroplasty in Medicare patients to 2040-2060. JB JS Open Access. 2023;8:e22.00112. 10.2106/JBJS.OA.22.00112. 36864906 10.2106/JBJS.OA.22.00112 PMC9974080

[2] Mohamed NS, Wilkie WA, Remily EA, Castrodad IMD, Jean-Pierre M, Jean-Pierre N, et al. The rise of obesity among total knee arthroplasty patients. J Knee Surg. 2020;35:1–6. 10.1055/s-0040-1710566. 32443160 10.1055/s-0040-1710566

[3] George J, Klika AK, Navale SM, Newman JM, Barsoum WK, Higuera CA. Obesity epidemic: is its impact on total joint arthroplasty underestimated? An analysis of national trends. Clin Orthop Relat Res. 2017;475:1798–806. 10.1007/s11999-016-5222-4. 28054327 10.1007/s11999-016-5222-4 PMC5449322

[4] Changulani M, Kalairajah Y, Peel T, Field RE. The relationship between obesity and the age at which hip and knee replacement is undertaken. The Journal of Bone and Joint Surgery British volume. 2008;90-B:360–3. 10.1302/0301-620X.90B3.19782. 10.1302/0301-620X.90B3.19782 18310761

[5] Ogden CL, Carroll MD, Kit BK, Flegal KM. Prevalence of childhood and adult obesity in the United States, 2011–2012. JAMA. 2014;311:806. 10.1001/jama.2014.732. 24570244 10.1001/jama.2014.732 PMC4770258

[6] Wallace G, Judge A, Prieto-Alhambra D, de Vries F, Arden NK, Cooper C. The effect of body mass index on the risk of post-operative complications during the 6 months following total hip replacement or total knee replacement surgery. Osteoarthritis Cartilage. 2014;22:918–27. 10.1016/j.joca.2014.04.013. 24836211 10.1016/j.joca.2014.04.013

[7] Hanly RJ, Marvi SK, Whitehouse SL, Crawford RW. Morbid obesity in total knee arthroplasty: joint-specific variance in outcomes for operative time, length of stay, and readmission. J Arthroplasty. 2017;32:2712–6. 10.1016/j.arth.2017.03.060. 28455175 10.1016/j.arth.2017.03.060

[8] Kremers HM, Visscher SL, Kremers WK, Naessens JM, Lewallen DG. The effect of obesity on direct medical costs in total knee arthroplasty. J Bone Joint Surg Am. 2014;96:718–24. 10.2106/JBJS.M.00819. 24806008 10.2106/JBJS.M.00819

[9] Carender CN, Glass NA, DeMik DE, Elkins JM, Brown TS, Bedard NA. Projected prevalence of obesity in aseptic revision total hip and knee arthroplasty. Iowa Orthop J. 2023;43:55–62. 37383860 PMC10296465

[10] D’Apuzzo MR, Novicoff WM, Browne JA. The John Insall Award: morbid obesity independently impacts complications, mortality, and resource use after TKA. Clin Orthop Relat Res. 2014;473:57. 10.1007/s11999-014-3668-9. 10.1007/s11999-014-3668-9 PMC4390915 24818736

[11] Prohaska MG, Keeney BJ, Beg HA, Swarup I, Moschetti WE, Kantor SR, et al. Preoperative body mass index and physical function are associated with length of stay and facility discharge after total knee arthroplasty. Knee. 2017;24:634–40. 10.1016/j.knee.2017.02.005. 28336148 10.1016/j.knee.2017.02.005 PMC5476206

[12] Schwarzkopf R, Thompson SL, Adwar SJ, Liublinska V, Slover JD. Postoperative complication rates in the “Super-Obese” hip and knee arthroplasty population. J Arthroplasty. 2012;27:397–401. 10.1016/j.arth.2011.04.017. 21676578 10.1016/j.arth.2011.04.017

[13] Workgroup of the American Association of Hip and Knee Surgeons Evidence Based Committee. Obesity and total joint arthroplasty: a literature based review. J Arthroplasty. 2013;28:714–21. 10.1016/j.arth.2013.02.011. 10.1016/j.arth.2013.02.011 23518425

[14] Wilson CD, Lundquist KF, Baruch NH, Gaddipati R, Hammonds KAP, Allen BC. Clinical pathways of patients denied total knee arthroplasty due to an institutional BMI cutoff. J Knee Surg. 2021;35:1364–9. 10.1055/s-0041-1723969. 33607678 10.1055/s-0041-1723969

[15] Riddle DL, Singh JA, Harmsen WS, Schleck CD, Lewallen DG. Clinically important body weight gain following total hip arthroplasty: a cohort study with 5-year follow-up. Osteoarthritis Cartilage. 2013;21:35–43. 10.1016/j.joca.2012.09.010. 23047011 10.1016/j.joca.2012.09.010 PMC4169300

[16] McElroy MJ, Pivec R, Issa K, Harwin SF, Mont MA. The effects of obesity and morbid obesity on outcomes in TKA. J Knee Surg. 2013;26:83–8. 10.1055/s-0033-1341407. 23479424 10.1055/s-0033-1341407

[17] Keys A, Fidanza F, Karvonen MJ, Kimura N, Taylor HL. Indices of relative weight and obesity. Int J Epidemiol. 2014;43:655–65. 10.1093/ije/dyu058. 24691951 10.1093/ije/dyu058

[18] Draft Guidance for Industry on Developing Products for Weight Management; Availability. Federal Register. 2007. https://www.federalregister.gov/documents/2007/02/15/E7-2581/draft-guidance-for-industry-on-developing-products-for-weight-management-availability. Accessed 22 Feb 2025.

[19] Konopka JA, Bloom DA, Lawrence KW, Oeding JF, Schwarzkopf RM, Lajam CM. Non-English speakers and socioeconomic minorities are significantly less likely to complete patient-reported outcome measures for total hip and knee arthroplasty: analysis of 16,119 cases. J Arthroplasty. 2023. 10.1016/j.arth.2023.01.005. 36682435 10.1016/j.arth.2023.01.005

[20] Bureau UC. New York State Population Topped 20 Million in 2020. Census.gov. https://www.census.gov/library/stories/state-by-state/new-york-population-change-between-census-decade.html. Accessed 16 Jan 2025.

[21] Hsieh FY, Bloch DA, Larsen MD. A simple method of sample size calculation for linear and logistic regression. Stat Med. 1998;17:1623–34 (10.1002/(sici)1097-0258(19980730)17:14%253C1623::aid-sim871%253E3.0.co;2-s). 9699234 10.1002/(sici)1097-0258(19980730)17:14<1623::aid-sim871>3.0.co;2-s

[22] Kim BI, Cochrane NH, O’Donnell JA, Wu M, Wellman SS, Ryan S, et al. Preoperative weight loss and postoperative weight gain independently increase risk for revision after primary total knee arthroplasty. J Arthroplasty. 2022;37:674–82. 10.1016/j.arth.2021.12.003. 34915131 10.1016/j.arth.2021.12.003

[23] Inacio MC, Silverstein DK, Raman R, Macera CA, Nichols JF, Shaffer RA, et al. Weight patterns before and after total joint arthroplasty and characteristics associated with weight change. Perm J. 2014;18:25–31. 10.7812/TPP/13-082. 24626069 10.7812/TPP/13-082 PMC3951027

[24] Seward MW, Chen AF. Obesity, preoperative weight loss, and telemedicine before total joint arthroplasty: a review. Arthroplasty. 2022;4:2. 10.1186/s42836-021-00102-7. 35005434 10.1186/s42836-021-00102-7 PMC8723914

[25] Haynes J, Nam D, Barrack RL. Obesity in total hip arthroplasty: does it make a difference? Bone Joint J. 2017;99-B(1 Supple A):31–6. 10.1302/0301-620X.99B1.BJJ-2016-0346.R1. 28042116 10.1302/0301-620X.99B1.BJJ-2016-0346.R1

[26] Goodnough LH, Finlay AK, Huddleston JI, Goodman SB, Maloney WJ, Amanatullah DF. Obesity is independently associated with early aseptic loosening in primary total hip arthroplasty. J Arthroplasty. 2018;33:882–6. 10.1016/j.arth.2017.09.069. 29089226 10.1016/j.arth.2017.09.069

[27] Bookman JS, Schwarzkopf R, Rathod P, Iorio R, Deshmukh AJ. Obesity: the modifiable risk factor in total joint arthroplasty. Orthop Clin North Am. 2018;49:291–6. 10.1016/j.ocl.2018.02.002. 29929710 10.1016/j.ocl.2018.02.002

[28] Carender CN, Glass NA, DeMik DE, Elkins JM, Brown TS, Bedard NA. Projected prevalence of obesity in primary total knee arthroplasty: how big will the problem get? J Arthroplasty. 2022;37:1289–95. 10.1016/j.arth.2022.03.003. 35271971 10.1016/j.arth.2022.03.003

[29] Hameed D, Bains SS, Dubin JA, Shul C, Chen Z, Stein A, et al. Timing matters: optimizing the timeframe for preoperative weight loss to mitigate postoperative infection risks in total knee arthroplasty. J Arthroplasty. 2024;39:1419-1423.e1. 10.1016/j.arth.2023.12.028. 38135167 10.1016/j.arth.2023.12.028

[30] Hales CM, Fryar CD, Carroll MD, Freedman DS, Ogden CL. Trends in obesity and severe obesity prevalence in US youth and adults by sex and age, 2007-2008 to 2015-2016. JAMA. 2018;319:1723–5. 10.1001/jama.2018.3060. 29570750 10.1001/jama.2018.3060 PMC5876828

[31] Phillips CL, Grayson BE. The immune remodel: weight loss-mediated inflammatory changes to obesity. Exp Biol Med (Maywood). 2020;245:109–21. 10.1177/1535370219900185. 31955604 10.1177/1535370219900185 PMC7016415

[32] Sax OC, Chen Z, Bains SS, Salib CG, Pervaiz SS, Mont MA, et al. Timing and type of bariatric surgery preceding total knee arthroplasty leads to similar complications and outcomes. J Arthroplasty. 2022;37:S842–8. 10.1016/j.arth.2022.01.076. 35121092 10.1016/j.arth.2022.01.076

[33] Jacofsky DJ, Springer BD, Mont MA, Ushakumari DS, Sladen RN. The impact of glucagon-like peptide-1 agonists on hip and knee arthroplasty and perioperative considerations. J Arthroplasty. 2024;39:1455–8. 10.1016/j.arth.2023.12.002. 38070716 10.1016/j.arth.2023.12.002

[34] Yoon H-K, Yoo J-H, Oh H-C, Ha J-W, Park S-H. The incidence rate, microbiological etiology, and results of treatments of prosthetic joint infection following total knee arthroplasty. J Clin Med. 2023;12:5908. 10.3390/jcm12185908. 37762849 10.3390/jcm12185908 PMC10532250

[35] Ayoade F, Li D, Mabrouk A, Todd JR. Periprosthetic Joint Infection. In: StatPearls. Treasure Island (FL): StatPearls Publishing; 2025. 28846340

[36] Jin X, Gallego Luxan B, Hanly M, Pratt NL, Harris I, de Steiger R, et al. Estimating incidence rates of periprosthetic joint infection after hip and knee arthroplasty for osteoarthritis using linked registry and administrative health data. Bone Joint J. 2022;104-B:1060–6. 10.1302/0301-620X.104B9.BJJ-2022-0116.R1. 36047015 10.1302/0301-620X.104B9.BJJ-2022-0116.R1 PMC9948458

[37] Gonzalez Della Valle A, Chiu YL, Ma Y, Mazumdar M, Memtsoudis SG. The metabolic syndrome in patients undergoing knee and hip arthroplasty: trends and in-hospital outcomes in the United States. J Arthroplasty. 2012;27:1743-1749.e1. 10.1016/j.arth.2012.04.011. 22677144 10.1016/j.arth.2012.04.011

